# Evaluating the effect and mechanism of upper limb motor function recovery induced by immersive virtual-reality-based rehabilitation for subacute stroke subjects: study protocol for a randomized controlled trial

**DOI:** 10.1186/s13063-019-3177-y

**Published:** 2019-02-06

**Authors:** Qianqian Huang, Wei Wu, Xiaolong Chen, Bo Wu, Longqiang Wu, Xiaoli Huang, Songhe Jiang, Lejian Huang

**Affiliations:** 1 The Second Affiliated Hospital and Yuying Children’s Hospital of Wenzhou Medical University, 109, Xueyuan W Road, Wenzhou, Zhejiang, 325027 China; 2 China-USA Neuroimaging Research Institute, the Second Affiliated Hospital and Yuying Children’s Hospital of Wenzhou Medical University, Wenzhou, Zhejiang, 325027 China; 3 Integrative & Optimized Medicine Research Center, China-USA Institute for Acupuncture and Rehabilitation, Wenzhou Medical University, Wenzhou, Zhejiang, 325027 China; 4 Department of Physiology, Northwestern University, Chicago, IL 60611 USA

**Keywords:** Immersive virtual reality training, Stroke, Upper extremity, Magnetic resonance imaging, Randomized controlled trial, Brain mechanism

## Abstract

**Background:**

There is compelling evidence of beneficial effects of non-immersive virtual reality (VR)-based intervention in the rehabilitation of patients with stroke, whereby patients experience both the real world and the virtual environment. However, to date, research on immersive VR-based rehabilitation is minimal. This study aims to design a randomized controlled trial to assess the effectiveness of immersive VR-based upper extremity rehabilitation in patients with subacute stroke and explore the underlying brain mechanisms of immersive VR-based rehabilitation.

**Methods:**

Subjects (*n* = 60) with subacute stroke (defined as more than 1 week and less than 12 weeks after stroke onset) will be recruited to participate in a single-blinded, randomized controlled trial. Subjects will be randomized 1:1 to either (1) an experimental intervention group, or (2) a conventional group (control). Over a 3-week time period immediately following baseline assessments and randomization, subjects in the experimental group will receive both immersive VR and conventional rehabilitation, while those in the control group will receive conventional rehabilitation only. During the rehabilitation period and over the following 12 weeks, upper extremity function, cognitive function, mental status, and daily living activity performance will be evaluated in the form of questionnaires. To trace brain reorganization in which upper extremity functions previously performed by ischemic-related brain areas are assumed by other brain areas, subjects will have brain scans immediately following enrollment but before randomization, immediately following the conclusion of rehabilitation, and 12 weeks after rehabilitation has concluded.

**Discussion:**

Effectiveness is assessed by evaluating motor improvement using the arm motor section of the Fugl-Meyer assessment. The study utilizes a cutting-edge brain neuroimaging approach to longitudinally trace the effectiveness of both VR-based and conventional training on stroke rehabilitation, which will hopefully describe the effects of the brain mechanisms of the intervention on recovery from stroke. Findings from the trial will greatly contribute to evidence on the use of immersive-VR-based training for stroke rehabilitation.

**Trial registration:**

ClinicalTrials.gov, NCT03086889. Registered on March 22, 2017.

## Background

Stroke is a major cause of death and long-term disability across the globe [1, 2] and its incidence is decreasing in the USA [3] but rising in China [4]. A common disabling consequence of stroke is upper limb dysfunction [5], which significantly affects patients’ activities of daily life. Therefore, one of the main goals of stroke rehabilitation is to improve upper limb function. Conventional rehabilitation techniques are effective in improving upper limb function but are resource-intensive and costly, often requiring specialized facilities not always widely available [6–8]. Moreover, in order to elicit significant improvement for stroke survivors, conventional upper limb rehabilitation usually requires 2–3 h of training per day for over 6 weeks [9], which is monotonous, confidence and interest draining for patients, and taxing on therapists. Therefore, it is imperative to find an alternative to overcome these drawbacks.

Virtual reality (VR)-based training might be one of the solutions. VR systems are classified as either immersive or non-immersive VR systems [10, 11]. In contrast to non-immersive VR systems, in which users experience both the real world and the virtual environment [10], immersive VR systems integrate users into an environment in which all real-world perception is blocked, so only computer-generated images are seen [10]. Similar to constraint-induced movement therapy (CIMT) [12, 13], and interactive video games [14], there is evidence that VR-wise interventions, which require repetitive and task-specific activities, could improve the restoration of upper limb function after stroke [15, 16]. Since VR is a type of interactive simulation combining computer hardware and software in which users can have close-to-reality experiences [17], it provides subjects with a more varied and realistic sensory perception experience and simulates the body movements of daily life, making rehabilitation more entertaining and involving for the subjects [18, 19].

Non-immersive VR systems have been widely used in stroke rehabilitation for several years, with the aim of improving motor function [6, 20]. Most of these studies have indicated that non-immersive VR-based rehabilitation is effective for upper limb functional improvement in individuals following stroke [21–24] but not significantly more beneficial compared with conventional rehabilitation, likely due to lack of relevant tasks provided by non-immersive VR [6]. Although it was reported that physical exercises through VR programs are effective for functional improvement in subjects with neurologic disorders [25] and that immersive VR systems may enhance motor learning and motor control [6], only one study has demonstrated that immersive VR-based rehabilitation can improve the effectiveness of fine hand-motion rehabilitation training [26]. However, what is the most appropriate frequency, intensity, and type of immersive VR-based rehabilitation to promote motor recovery and critical brain reorganization in this early post-stroke stage remains unknown. Functional magnetic resonance imaging (fMRI) has been important in exploring the neural mechanisms of recovery after brain disease, for instance, exploring patients’ motor execution networks post-stroke [27, 28]. Therefore, a longitudinal magnetic resonance image (MRI) study (similar to the ones performed previously in subjects with subacute back pain [29, 30]) will be implemented. Using MRI techniques, brain mechanisms related to strategies that enable the rehabilitation of repetitive, relevant, and skilled activities in the early stage post-stroke can be explored.

There are two reasons for choosing subjects with subacute stoke rather than other stages post-stroke in the clinic trial. First, in this subacute stage, patients have been shown to have the best and most rapid functional recovery [31], which benefits the observations of critical brain reorganization. Second, in China most stroke patients would be hospitalized during this stage, which makes recruitment and MRI scanning easier.

In summary, in this study we will use immersive VR training in post-stroke rehabilitation and assess the effectiveness in eliciting upper-limb motor recovery post-stroke compared to traditional rehabilitation training. Additionally, we will investigate the underlying brain mechanisms of immersive VR-based rehabilitation using MRI techniques.

## Methods/design

### Aim

The aim of the study is to assess the effectiveness of immersive VR-based upper extremity rehabilitation on patients with subacute stroke and explore the underlying brain mechanisms of immersive VR-based rehabilitation. The arm motor section of the Fugl-Meyer assessment (FMA) [32, 33] will be used to assess the effectiveness, and MRI techniques will be applied to investigate the brain mechanisms. The details will be discussed in the sections “Primary outcome measure” and “Secondary outcome measures”. It is presumed that motor learning and motor control is pivotal in the development of sensorimotor interventions for post-stroke recovery and that VR-based intervention may enhance motor learning and motor control [6].

### Study design

The Institutional Review Board of the Second Affiliated Hospital and Yuying’s Children Hospital, Wenzhou Medical University, China, approved this study. Prior to inclusion, all subjects will be informed about the objectives and procedures of the study. Subjects who meet the inclusion criteria must provide informed consent before entering into the study.

Subjects (*n* = 60) diagnosed with stoke in its subacute stage (defined as more than 1 week and less than 12 weeks after stroke onset) from an in-patient stroke rehabilitation unit in China will be enrolled in a single-blinded randomized controlled trial for 15 weeks. They will be randomized in a 1:1 fashion into (1) a new 3-week rehabilitation training program with an immersive VR system, (2) a 3-week conventional rehabilitation program. The random allocation will follow a covariate-adaptive randomization procedure [34, 35]. Each subject will be randomly assigned a code based on computer-generated, stratified, permuted block randomization with a block size of 8 and balanced by age and location of stroke.

**Fig. 1:**
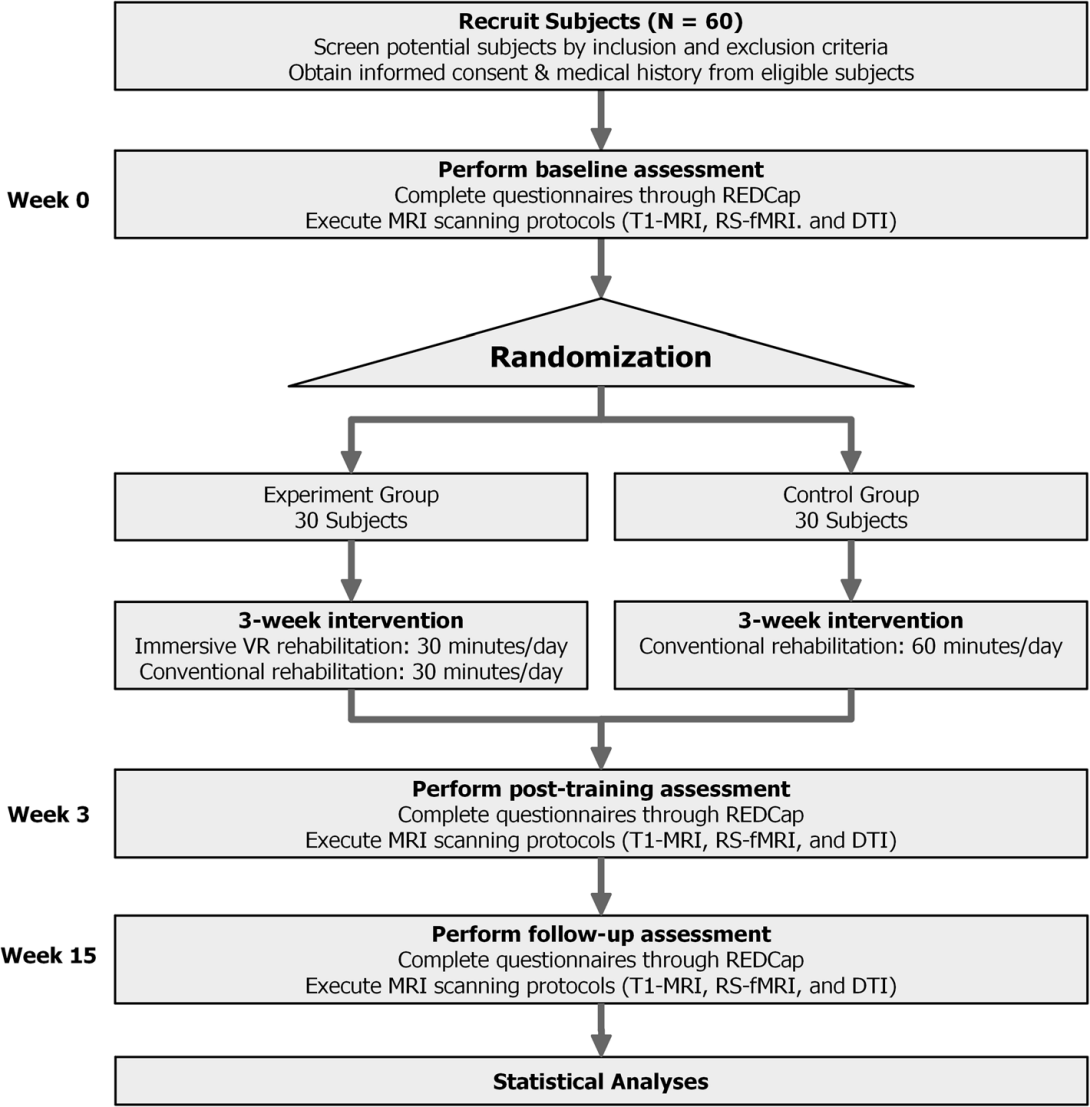
Chart of study flow. REDCap, Research electronic data capture; T1-MRI, high-resolution anatomical magnetic resonance imaging; RS-fMRI, resting-state functional magnetic resonance imaging; DTI, diffusion tensor imaging; VR, virtual reality

**Fig. 2:**
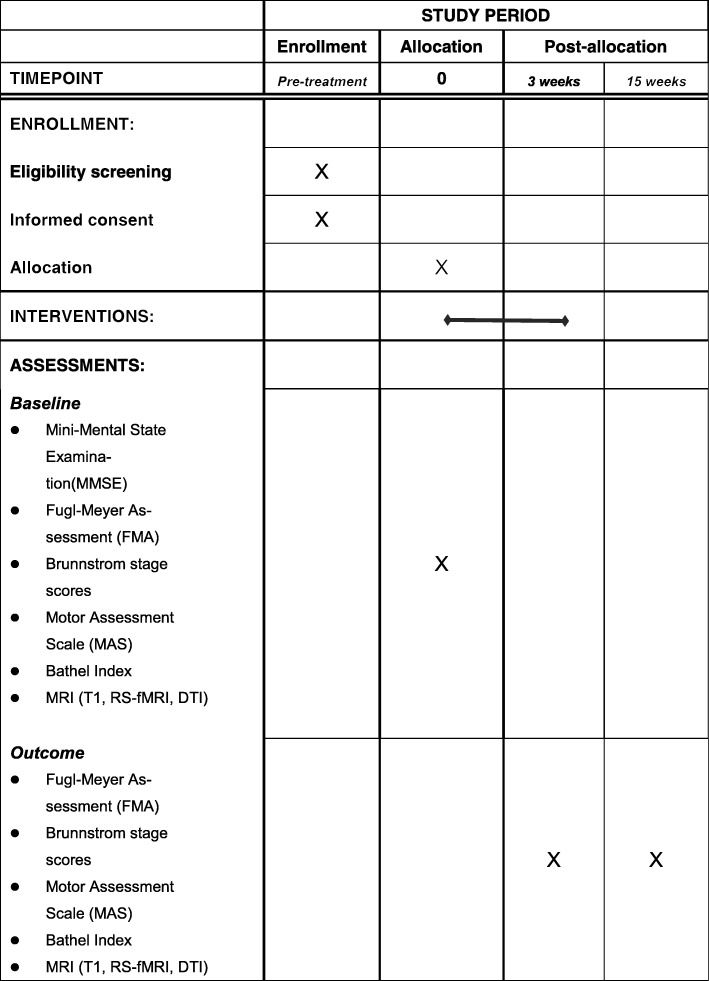
Schedule of enrollment, interventions, and assessments of the study. MRI (T1, RS-fMRI, DTI), magnetic resonance imaging (high-resolution anatomical, resting-state functional MRI, diffusion tensor imaging)

All subjects will receive three assessments at the following time points: immediately following enrollment but before randomization (week 0), immediately following conclusion of the randomized rehabilitation program (week 3) and follow up 12 weeks after conclusion of the rehabilitation program (week 15). The assessment includes inclusion and exclusion of subjects, clinician-filled questionnaires and MRI scans. To avoid assessment bias, all inclusion and exclusion assessments and clinician-filled questionnaires will be completed by physiotherapists who are blinded to this study and with at least 2-years of experience in physical therapy. The flow chart for the study is shown in Fig. 1 and the schedule of enrollment, interventions, and assessments of the study (as recommended by Standard protocol items: recommendation for interventional trials (spirit) 2013 [36]) is shown in Fig. 2.

### Participants

The inclusion criteria are as follows: to be eligible, subjects must (1) be over 30 but less than 85 years old; (2) have had their first stroke within the past month; (3) have a diagnosis of acute stroke confirmed by a neuroimaging neurological assessment (computed tomography or MRI); (4) have a starting upper-limb function of Brunnstrom [37] stage II~IV; and (5) have good cognitive ability (Mini-Mental State Examination (MMSE) [38] cutoff > 23).

The exclusion criteria are as follows: (1) history of transient ischemic attack (TIA); (2) failure of critical organs, such as heart, lung, liver, and kidney; (3) previous history of brain neurosurgery or epilepsy; (4) severe cognitive impairments or aphasia (incapable of understanding the instructions given by therapists); (5) not suitable for an MRI scan (including but not limited to: metal fragments in eyes or face; implantation of any electronic devices such as (but not limited to) cardiac pacemakers, cardiac defibrillators, cochlea implants or nerve stimulators; surgery on the blood vessels of brain or the valves of the heart; claustrophobia; or brain or skull abnormalities); (6) life expectancy < 3 months; and (7) enrollment in another clinical trial involving physical therapy or an investigational drug.

### Sample size considerations

This randomized controlled trial is a two-group independent design examining the effects of immersive VR on rehabilitation of subjects with subacute stroke. We assumed a two-tailed comparison and set the type I error rate at 0.05 with 80% power. We plan to screen approximately 100 individuals with subacute stroke. After screening, 80 subjects will be recruited and randomized to the experimental group or the control group. As a conservative estimate (dropout rate = 25%), we presume that 60 subjects will complete the study. To reduce the dropout rate, we will employ two strategies to keep participants engaged: regular communication via phone or social media and clinician visits. After conducting a power analysis based on the aforementioned statistical parameters using the software GPower3.1.9.2 [39], the effect size is calculated as 0.74, which is between a medium (0.5) and large (0.8) effect size [40]. Moreover, there is evidence from a small sample (8 subjects) that VR-enhanced treadmill training for 5 sessions per week over 3 weeks induces significant cerebral reorganization [41]. Thus, 30 subjects for one group is sufficient to assess the effectiveness of immersive VR training in post-stroke rehabilitation in eliciting upper-limb motor recovery post-stroke compared to traditional rehabilitation training, and to investigate the underlying brain mechanisms of immersive VR-based rehabilitation using three MRI modalities.

### Intervention design

Subjects in the control group will receive 60-min conventional rehabilitation training per day, 5 days per week for 3 weeks. This conventional rehabilitation delivered by a therapist at the hospital includes physical and occupational therapy (upper extremities flexion and extension training) which comprise task-related practice for gross movements and dexterity, including different grips and selective finger movements, strength training, stretching, and training in daily life activities. Conventional rehabilitation will be designed with similar intensity and complexity to simulate the skills required in the immersive VR group. Researchers in the study will supervise and encourage all participant to fully participate in the training to guarantee the quality of the training. In contrast, subjects in the experimental group will receive 30 min of conventional rehabilitation and 30 min of immersive VR rehabilitation training per day, 5 days per week for 3 weeks conducted by a therapist at the hospital. The details will be discussed in the section “VR training protocol”.

### VR system

**Fig. 3:**
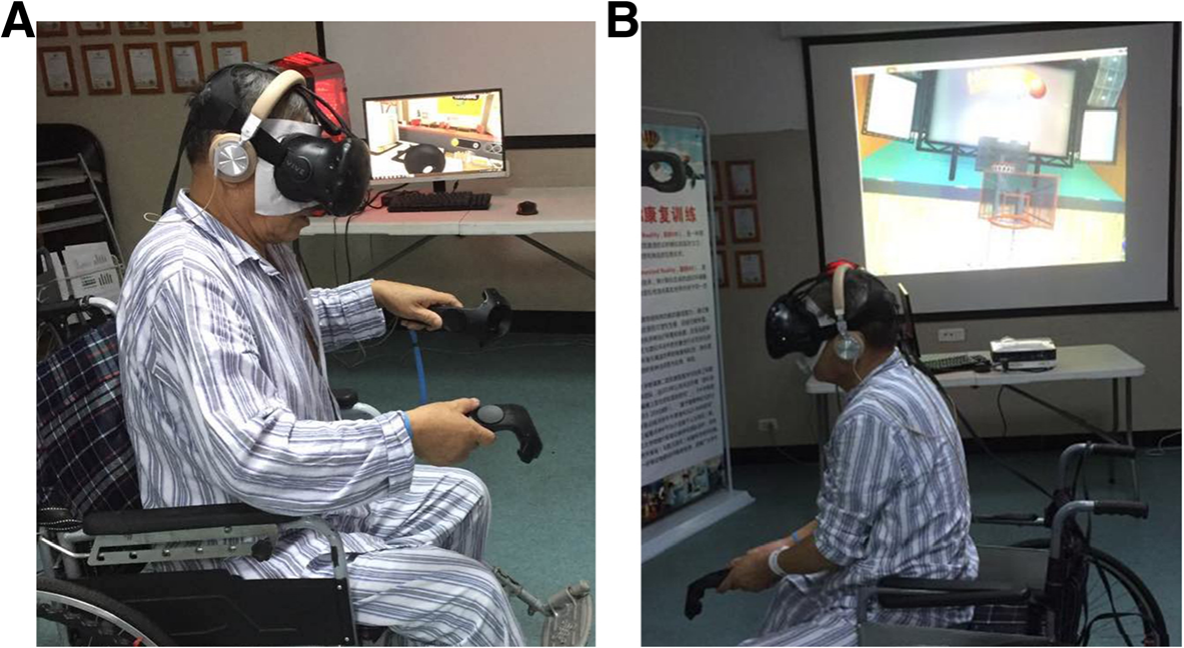
Virtual reality (VR) system and a virtual scenario. **a** A subject is wearing a VR system. **b** A subject is playing basketball

Shown in Fig. 3a, the VR system consists of (1) a head-mounted display (HMD) (HTC Vive-VR); (2) a pair of wireless controllers; (3) two base stations, where steamVR® tracking technology tracks a subject’s exact location and movement through the headset and controllers; (4) a computer with a 4-core Intel® Core™ i5–4590 at 3.30 GHz, 8-GB random access memory (RAM), and a NVIDIA® Geforce® GTX 1070/MSI with 8 GB of GDDR5. Wearing the HMD and sitting in a wheelchair, subjects in the experimental group will interact with virtual objects in various scenarios.

### VR training protocol

**Fig. 4:**
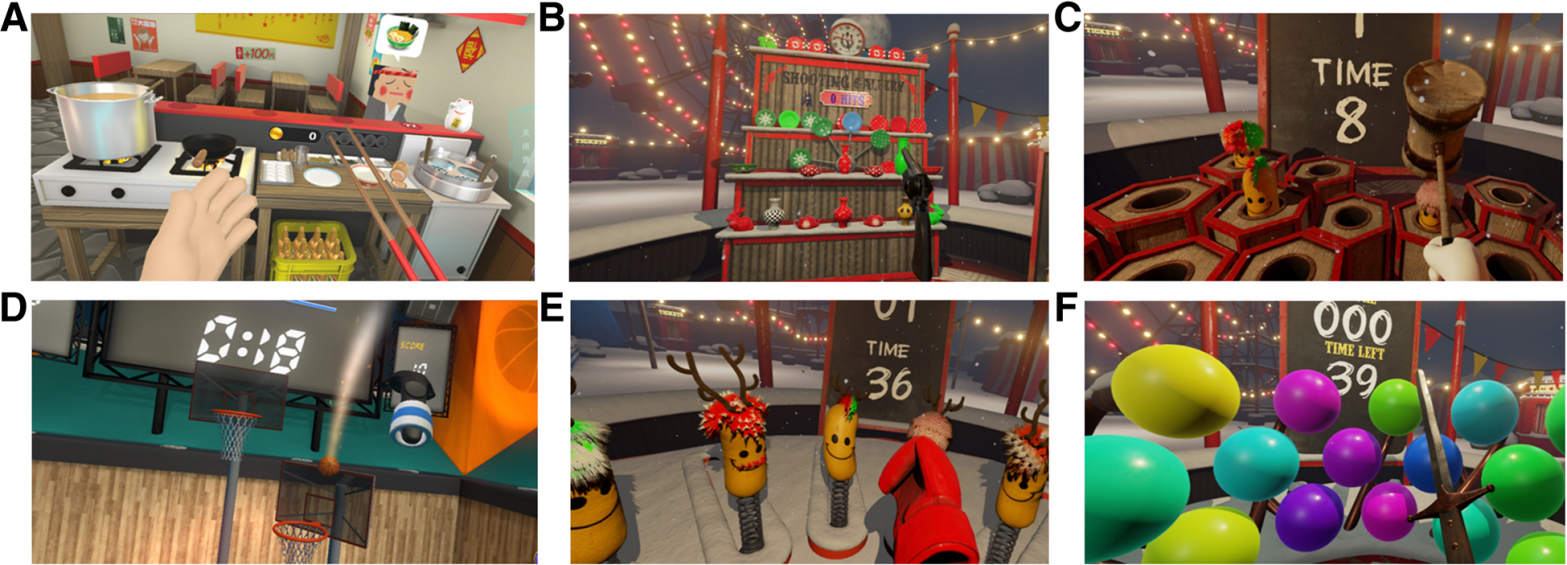
Six virtual reality (VR) programs. **a** Making scrambled eggs and frying dumplings in a virtual kitchen by controlling a hand and a pair of chopsticks, respectively. **b** Shooting ceramic plates and vases on a shelf by controlling a pistol in a virtual shooting gallery. **c** Playing a whack-a-mole game by controlling a wooden mallet hammer in a virtual playground. **d** Playing basketball in a virtual court, in which the ball is shot by a controller and the height and distance of the basket is varied over time. **e** Punching with dolls by controlling a big fist in a virtual boxing arena, in which the doll that is hit will retreat to its original position. **f** Popping balloons by controlling a sword in a virtual fencing hall

During the daily 30-min VR rehabilitation, subjects in the experimental group will be required to complete the six programs (kitchen, shooting gallery, playground, basketball court, boxing arena, fencing hall) shown and described in Fig. 4, playing different roles in a virtual environment. When using the HTC Vive-VR HMD, the patient can look around by physically turning the head to a limited degree.

This is close to a realistic experience and provides a high level of immersion. In the early stages of rehabilitation, due to the poor function of the upper limb on the hemiplegic side, subjects will have to complete the VR programs with the help of the limb on the unaffected side. With the recovery of upper limb function on the hemiplegic side, the subjects will then independently complete the six games using the limb on the hemiplegic side.

### Primary outcome measure

The arm motor section of the Fugl-Meyer assessment (FMA) [32, 33], highly recommended as a clinical tool for evaluating changes in motor impairment after stroke [33], will be used to measure arm movement ability across several domains: motor function, balance, sensation, range of motion, and pain.

### Secondary outcome measures

We will apply MRI techniques to assess the performance of immersive VR-based rehabilitation and to investigate the brain mechanisms of rehabilitation, particularly in regions related to motor learning and motor control. Three different MRI modalities will be acquired: (1) high-resolution anatomical MRI (T1-MRI) for estimating anatomical parameters of cortex, subcortex, and structural connectivity; (2) resting-state functional MRI (RS-fMRI) for estimating the functional connectivity of brain regions; and (3) diffusion tensor imaging (DTI) for estimating tractographic parameters of white matter and the microstructure of gray matter. Each subject will be scanned three times (before rehabilitation, immediately following 3 weeks of rehabilitation, and 12 weeks post-rehabilitation). The brain parameters obtained by MRI will be assessed and correlation with the clinical outcome measures will be tested.

Other secondary outcome measures include: (1) Brunnstrom stage scores, which will be obtained for assessment of motor recovery of the upper extremities; (2) assessment of the subject’s’ mental state, which will be determined using the Hamilton Depression Scale (HAMD) [42] and the Hamilton Anxiety Scale (HAMA) [43]; (3) the activities of daily living (ADL), which will be determined using the Function Independent Measure (FIM) [44] and the Bathel Index (BI) [45]; (4) assessment of the severity of ischemic stroke and cognitive function, which will be determined using the National Institutes of Health Stroke Scale (NIHSS) [46] and the Korea-Mini Mental Status Evaluation (K-MMSE) [38], respectively. These stoke-associated measures will also be evaluated as covariates in regard to the primary outcome measure.

### Questionnaire data acquisition

All questionnaires will be collected on an electronic tablet on which the questionnaire software, Research Electronic Data Capture (REDCap), will be installed [47]. REDCap is a secure, convenient, and efficient online/offline Web application for capturing electronic survey data and is recommended by the National Institutes of Health for data collection in clinical trials.

### MRI data acquisition

Subjects will be scanned on a 3-T GE-Discovery 750 scanner at Wenzhou Medical University (WMU), China, equipped with the following: for anatomical T1-MRI data, repetition time (TR)TR/echo time (TE) = 7.7/3.4 ms, flip angle = 12°, field of view (FOV) = 256 mm × 256 mm, resolution = 256 × 256, slice per volume = 176, slice thickness = 1 mm; for RS-fMRI data, TE/TR = 30/2500 ms, voxel size = 3.4375 × 3.4375 × 3.5 mm^3^, in-plane resolution = 64 × 64, number of volumes = 230, and flip angle = 90°; and for DTI data, TR/TE = 8000/80 ms, flip angle = 90°, FOV = 256 mm × 256 mm, resolution = 128 × 128, slice thickness = 2 mm, slices per volume = 75, 23 volumes with b = 1000 s/mm^2^ and 49 volumes with b = 2000 s/mm^2^.

### MRI data processing

Data quality control: each imaging modality (T1, RS-fMRI and DTI) will undergo quality control and data with excessive motion or bad signal-to-noise ratio will be excluded [48]. Three different metrics are evaluated for each subject: (1) for T1, the volume of seven regions of interest (ROIs) and the stretch factor (transformation required to move the native-space in the brain to template space) is obtained; (2) for RS-fMRI, the temporal signal-to-noise ratio is calculated; and (3) for DTI, the mean fractional anisotrophy (FA) and mean diffusivity is obtained for specific ROIs and for white matter. These metrics must be met for the obtained scans to be accepted and to start the analysis for each modality.

The subjects’ structural T1 images will be analyzed using the FSL subcortical segmentation tool “FIRST” [49] and with the FSL gray matter density tool “VBM” [50]. From the results of FIRST, which includes 14 subcortical regions, and structural connectivity that correlates in 360 parcellations of cerebral cortex [51], we will find ROIs that may correlate with rehabilitation performance and continuing performance. Meanwhile, from the VBM result, the significant difference in local gray matter regions will be evaluated.

The subject’s RS-fMRI will be analyzed using FSL tools. First, we will use an independent component analysis (ICA)-based strategy for automatic removal of motion artifacts (AROMA) [52] to pre-process the data and correct resting images for motion. Second, we will regress out any signal found in ventricles and in white matter as these are considered artifacts. We will also remove the whole-brain global signal. To allow comparison between subjects, these filtered functional data will be registered to a standard brain and down-sampled to a 6 × 6 × 6 mm voxel size. A program produced in house called Apkarian Brain Linkage Mapping (ABLM), described and reported by Baria [53], will be used to provide the number of functional connectivity links of each brain voxel.

The subject’s DTI will be analyzed using FSL tools. We will use the diffusion toolbox “FDT” [54] to pre-process the DTI images. First, we will correct each subject’s DTI images for “eddy current” and then apply a “dtifit” algorithm to obtain a diffusion tensor model value at each white matter voxel. We will further extract the mean FA value of a group of voxels that may identify regions with significantly different white matter fiber microstructure [55, 56].

### Statistical analysis

We will use the IBM SPSS Statistics 24 [57] and R [58] to perform statistical analyses. Two-way repeated measures analysis of variance (ANOVA) will be used to compare the group effect. If subjects are lost to follow up, an intention-to-treat analysis will be conducted. Student’S *t* test will be used to compare the within-group changes when ANOVA reveals a significant difference. Statistical significance is determined by a two-sided *p* value of less than 0.05. In addition, if any demographic or clinical characteristics are significantly imbalanced, analysis of covariance will be performed to adjust the imbalance. To help prevent missing data, all questionnaires are user friendly and collected electronically, and all personnel related to the study are trained to identify and engage the subjects who are at the greatest risk of dropout during follow up. In cases of missing data, the method of baseline observation carried forward [59] will be used to impute these data from all enrolled subjects.

### Study oversight and participant confidentiality

Study oversight will be under the direction of an Independent Safety Monitoring Board (ISM), who is composed of experts in clinical trials, medical ethics, statistics, and data management. The ISM is independent of the study and the sponsor, and is responsible for monitoring data and participant safety.

We are committed to respecting participant privacy and to keep personal information confidential. All participants’ personal and health information and brain imaging data will be maintained on a secure server. Access to these data are password protected, and all data are anonymized and coded prior to uploading to the server.

## Discussion

This clinical trial aims to evaluate the effectiveness of immersive VR-based rehabilitation in upper-limb motor recovery post-stroke compared to traditional rehabilitation through questionnaires and evaluation of brain mechanisms via MRI. Previous studies have shown that non-immersive VR is safe but not significantly more beneficial compared with conventional rehabilitation [6]. It was suggested, however, that immersive VR systems may have more beneficial results in improving the patient’s upper extremity motor function and quality of life after stroke, as these VR system programs provide a combination of extra spatial transformation of uncoupled eye–hand movements, enhanced motion control, and more entertainment for patients, thereby motivating motion learning [6]. The results of this study will provide evidence to either support or not support this hypothesis.

To avoid creating an imbalance between the two groups in rehabilitation time, which may introduce additional variables to explain the resultant improvement in upper extremity motor function, the total rehabilitation time is set to be identical for the two groups. Moreover, in order to observe the long-term outcomes of immersive VR-based rehabilitation in terms of upper limb motor function, the trial will follow up all subjects 12 weeks after they have finished rehabilitation.

The main limitation in this trial is its single-center design and the limited sample size. There is clearly a need for multicenter, large-scale trials to determine the benefits of immersive VR-based rehabilitation, but before launching these it is important to demonstrate feasibility and effectiveness. Another limitation is that current immersive VR technology is still unable to simulate all rehabilitation training. As a compromise, the subjects in the experimental group will experience a combination of 30-min normal rehabilitation and 30-min immersive VR rehabilitation.

In conclusion, this study aims to explore the immediate and longer-term effects of immersive VR-based rehabilitation in subjects in the early stage of stroke, and to discuss the mechanism of its impact on the brain’s anatomical and functional reorganization. The results of the trial will be of benefit to future patients with stroke and may provide a new and better method of stroke rehabilitation.

## Trial status

Subject recruitment is underway.

## Abbreviations

ABLM: Apkarian Brain Linkage Mapping
ADL: Activities of daily living
ANOVA: Analysis of variance
AROMA: Automatic removal of motion artifacts
BI: Bathel Index
DTI: Diffusion tensor image
FMA: Fugl-Meyer assessment
fMRI: Functional magnetic resonance imaging
FOV: Field of view
HAMA: Hamilton Anxiety Scale
HAMD: Hamilton Depression Scale
HMD: Head mounted display
ISM: Independent safety monitor
K-MMSE: Korea-Mini Mental Status Evaluation
RCT: Randomized controlled trial
REDCap: Research electronic data capture
ROI: Region of interest
RS-fMRI: Resting-state functional magnetic resonance imaging
T1-MRI: High-resolution anatomical magnetic resonance imaging
TIA: Transient ischemic attack
TE: Echo time
TR: Repetition time
VR: Virtual reality
WMU: Wenzhou Medical University
